# Preparation of Ethosome Gel with Total Flavonoids from *Vernonia anthelmintica* (L.) Willd. for the Treatment of Vitiligo

**DOI:** 10.3390/gels11010073

**Published:** 2025-01-17

**Authors:** Dongmei Qin, Yongjie Cui, Mengyue Zheng, Zhiguo Yang, Xinbing Wang

**Affiliations:** Key Laboratory of Xinjiang Phytomedicine Resource and Utilization, Ministry of Education, School of Pharmacy, Shihezi University, Shihezi 832002, China; 15039826785@163.com (Y.C.); 13579761260@163.com (X.W.)

**Keywords:** *Vernonia anthelmintica* (L.) Willd., ethosome, Carbomer 934 gel, Vitiligo

## Abstract

*Vernonia anthelmintica* (L.) Willd. is a traditional medicinal herb in Chinese medicine, extensively used by various ethnic groups due to the numerous advantages derived from its total flavonoids. These benefits encompass anti-inflammatory and antioxidant effects, and the promotion of melanin production, showcasing its significant efficacy in addressing vitiligo. To improve transdermal absorption and enhance the antioxidant effectiveness of the treatment, ethosome containing total flavonoids were prepared utilizing the ultrasound injection technique. The resulting ethosome was then carefully mixed with 0.7% Carbomer 934 gel in equal parts, yielding a gel concentration of 0.302 mg/g. This formulation produced small, consistent ethosome that exhibited high encapsulation efficiency and notable stability. In vitro analyses demonstrated sustained release characteristics of the gel and considerable therapeutic effectiveness against vitiligo resulting from hydroquinone exposure. Histological examinations performed through hematoxylin and eosin (H&E) staining of mouse skin revealed increased melanin production and increased activities of tyrosinase (TYR), cholinesterase (CHE), and mouse monoamine oxidase (MAO), while levels of superoxide dismutase (SOD) and malondialdehyde (MDA) were reduced. These findings underscore the promising effectiveness of this treatment strategy and validate the efficacy of the dosage form.

## 1. Introduction

Vitiligo is a common skin disorder characterized by depigmentation, impacting approximately 0.5% to 2% of people globally [1]. The prevalence of this condition is relatively similar in both genders. A defining feature of vitiligo is the targeted loss of melanocytes, which frequently manifests on the skin as depigmented spots of various shapes and sizes, which may be round or irregular [2]. Areas affected by vitiligo are often located on the head, face, and at the borders between skin and mucous membranes, which can significantly influence an individual’s appearance [3]. As a consequence, this disorder may impose a substantial psychological burden on those affected, detrimentally impacting their quality of life. Therefore, investigations into the causes, prevention strategies, and treatment options for vitiligo have remained a key area of interest for medical researchers around the globe. Key elements in the development of vitiligo include oxidative stress and tyrosinase, which play significant roles in the disease’s pathological mechanisms [4]. Tyrosinase is an essential enzyme for melanin production and typically facilitates the oxidation polymerization of tyrosine to generate melanocytes, which are crucial for maintaining normal skin pigmentation [5].

In contrast, individuals suffering from vitiligo show a reduced activity of tyrosinase, leading to a decreased capacity of melanocytes to produce melanin, thereby forming white patches on their skin. Studies have shown that oxidative stress triggers free radical reactions that can either directly or indirectly reduce tyrosinase functionality, leading to cellular damage [6].

On the one hand, oxidative stress increases the production of intracellular free radicals. Excessive free radicals can damage DNA or proteins involved in tyrosinase synthesis within cells, thereby directly reducing the tyrosinase synthesis enzyme and impairing the repair mechanisms [7]. On the other hand, free radical reactions induced by oxidative stress may also diminish enzyme activity by altering the substrate environment of tyrosinase [8]. For instance, oxidative stress can lead to the abnormal aggregation of precursor molecules essential for melanin synthesis, resulting in the formation of oxidized polymers that may reduce the catalytic efficiency of tyrosinase and subsequently impair normal melanin production [9,10]. Therefore, the clinical treatment of vitiligo focuses on reducing oxidative damage and enhancing tyrosinase activity [11].

Currently, the primary strategies for managing vitiligo include topical therapies, phototherapy, oral treatments, and surgical interventions [12]. Topical therapy represents a crucial modality in treating skin disorders [13,14]. The clinical use of transdermal drug delivery has gained popularity as a reliable and effective method [15]. The effectiveness of topical formulations is significantly affected by the specific type and properties of the medication. Various topical agents, including corticosteroids, non-steroidal anti-inflammatory medications, photosensitizers, and vitamin D_3_, are employed in treating vitiligo; however, they frequently yield unsatisfactory results, with a notable risk of recurrence. Furthermore, these therapies can provoke skin irritation, trigger allergic responses, and worsen the condition [16,17]. The formulation type also plays a vital role in determining drug effectiveness. Commonly used topical forms for treating vitiligo are ointments and creams, which provide advantages such as convenience and portability. Nevertheless, their ability to be absorbed through the skin is often restricted, and an excess of excipients might lead to negative side effects, including allergies. Hence, it is clear that topical therapy faces significant obstacles in the management of vitiligo. As a result, it is crucial to identify safe and effective therapeutic agents and to create appropriate formulations to improve the overall effectiveness of vitiligo treatment.

*Vernonia anthelmintica* (L.) Willd. serves as a commonly used traditional medicine among the Uyghur people for treating vitiligo [18]. Studies exploring the phytochemical composition of *Vernonia anthelmintica* (L.) Willd., recognized in traditional Chinese medicine for its effectiveness against vitiligo, have revealed various compounds, including phenolic acids, chalcones, flavonoids, terpenes, fatty acids, and steroids. Among these, flavonoids such as butein, luteolin, and butin are noted for their ability to modulate melanin synthesis and bolster immune responses, demonstrating considerable antioxidant and anti-inflammatory properties [19,20]. As a result, flavonoids are considered the primary active components of *Vernonia anthelmintica* (L.) Willd. in combating vitiligo. Different techniques, including microneedles and penetration enhancers, have been utilized to enhance the transdermal drug delivery. Nevertheless, these methods present certain limitations, such as instability and potential complications arising from their formulation.

Ethosome are a type of vesicular system comprising phospholipids, water, Carbomer 934, and high concentrations of low molecular weight alcohols [21]. They maintain the traditional targeting abilities and favorable biocompatibility associated with ethosome, while simultaneously enhancing drug penetration through the skin, boosting drug accumulation at the lesion location, and providing improved therapeutic outcomes [22]. Based on previous studies, this research incorporated purified flavonoids derived from the extract of *Vernonia anthelmintica* (L.) Willd. into ethosome. To further prolong the retention time on the skin and extend drug efficacy, this study investigated embedding ethosome within a gel system. This method not only improves the stability of the ethosomal formulation but also assists in retaining the drug on the skin surface. Furthermore, the formulation’s effectiveness for treating vitiligo was assessed in hydroquinone-induced vitiligo mice models.

## 2. Results and Discussion

### 2.1. Screening of Ethosome Preparation Methods

The methods of injection-ultrasound and thin film dispersion were examined and assessed. Both forms of ethosome, upon preparation, manifested as clear liquids displaying a pale blue opalescence, demonstrating the Tyndall effect. The ethosome formed through the injection-ultrasound technique had a measured particle size of 77.97 ± 0.57 nm and a polydispersity index (PDI) of 0.241 ± 0.007. In contrast, those obtained from the thin film dispersion method showed a larger particle size of 108.91 ± 0.76 nm and a PDI of 0.308 ± 0.006. After a storage period of two weeks at 4 °C, the ethosome created by the thin film dispersion method displayed clumping and sedimentation, highlighting instability within the system. In comparison, the ethosome generated through the injection-ultrasound technique showed no significant changes. As a result, the findings of this study highlighted the injection-ultrasound method as the more favorable technique for ethosome preparation.

### 2.2. Selection of the Alcohol Phase of Ethosome

**Figure 1:**
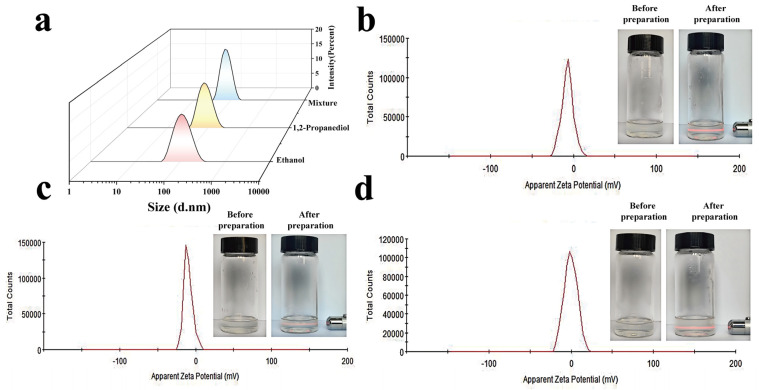
Appearance and characterization of three ethosomes. (**a**) Particle size distribution of three ethosomes. (**b**) Appearance and zeta potential distribution of ethanol ethosome. (**c**) Appearance and zeta potential distribution of 1,2-propanediol ethosome. (**d**) Appearance and zeta potential distribution of mixture ethosome.

The characteristics of blank ethosome created with various alcohol types, including particle size, PDI, and zeta potential, are summarized in Table 1. Figure 1 demonstrates the visual properties of the three ethosome types, along with their respective particle size and zeta potential distributions. The findings reveal that all three ethosome variations appeared clear and transparent, producing a distinct light path when exposed to a beam, highlighting the Tyndall effect. Mixtures were formulated using a combination of ethanol and 1,2-propanediol, as this particular alcohol phase displayed superior clarity. The particle size and zeta potential analyses for the three ethosome types indicated consistent particle size distributions (PDI < 0.3) and minimal zeta potentials, implying a comparatively stable formulation that resisted aggregation. The ethosome in the mixed alcohol system showed smaller particle sizes and a more consistent distribution than the other ethosome types. Therefore, this investigation adopted a blend of ethanol and 1,2-propanediol as the alcohol phase for further ethosome production and analysis.

### 2.3. Prescription Screening of Total Flavonoid Ethosome

#### 2.3.1. Effect of the Cholesterol/Soy Lecithin Ratio on Total Flavonoid Ethosome

Cholesterol, a type of steroid that comprises a sterol component and an extended hydrocarbon chain, is essential for modulating the fluidity and permeability of phospholipid bilayers. Cholesterol is frequently referred to as a “fluidity buffer” [23] due to its capacity to alter the molecular configuration of lipid membranes in response to variations in temperature. This adaptation helps maintain membrane flexibility, increases the resistance of the phospholipid bilayer to external influences, and minimizes the risk of drug leakage. As a result, this adjustment enhances the encapsulation efficiency of the drug. Research indicates that moderate cholesterol levels can bolster the phospholipid bilayer’s stability. Nevertheless, excessive cholesterol can disrupt the optimal cholesterol/soy lecithin ratio, which may jeopardize the structural integrity of the bilayer. Such an imbalance can lead to drug leakage and reduced encapsulation rates [24,25]. Therefore, determining the ideal cholesterol/soy lecithin ratio is vital for enhancing the stability of ethosome.

**Figure 2:**
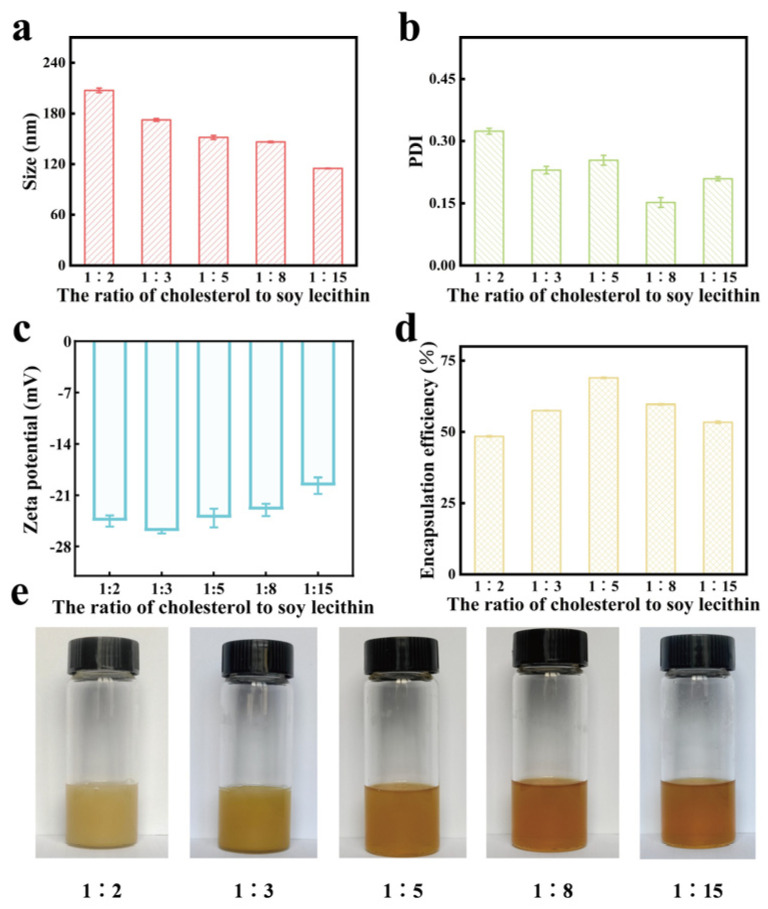
Effect of cholesterol/lecithin ratio on total flavonoid ethosome. (**a**) Effect of cholesterol/lecithin ratio on size of total flavonoid ethosome. (**b**) Effect of cholesterol/lecithin ratio on PDI of total flavonoid ethosome. (**c**) Effect of cholesterol/lecithin ratio on zeta potential of total flavonoid ethosome. (**d**) Effect of cholesterol/lecithin ratio on encapsulation efficiency of total flavonoid ethosome. (**e**) Appearance of total flavonoid ethosome with different cholesterol/lecithin ratio.

The ethosome loaded with the drug, created using different ratios of cholesterol/soy lecithin, are depicted in Figure 2. This figure shows that ethosome made with cholesterol/soy lecithin ratios of 1:2 or 1:3 presented a milky-white coloration. In contrast, ethosome formulated with lower ratios revealed a more consistent and clear quality. Figure 2 illustrates how varying proportions of cholesterol/soy lecithin influence the characterization and encapsulation efficiency of ethosome containing extract from *Vernonia anthelmintica* (L.) Willd. seed. An increase in cholesterol content within the mixture resulted in a corresponding increase in both the particle size and the polydispersity index (PDI) of the ethosome. At a cholesterol/soy lecithin ratio of 1:5, the ethosome exhibited the highest encapsulation efficiency, demonstrating a uniform distribution of particle sizes (PDI < 0.3) and a zeta potential of approximately −25 mV, indicating stability within the system. Consequently, the 1:5 cholesterol/soy lecithin ratio was selected for further investigation.

#### 2.3.2. Effect of the Alcohol Phase Ratio on Total Flavonoid Ethosome

The alcohol phase is a critical component of the ethosome system, significantly influencing the permeability of the ethosome formulation [26]. Alcohol molecules play a vital role in the formation of lipid bilayer in ethosome; their incorporation can reduce the ethosome membrane’s thickness and enhance the ethosome structure’s fluidity, thereby improving the skin penetration of drugs and markedly increasing transdermal drug delivery. Furthermore, alcohol molecules possess a certain degree of negative charge, which contributes to a higher surface charge of the ethosome formulation, enhancing the physical stability of the ethosome and rendering the formulation more robust. Additionally, lipophilic and amphiphilic drugs are more effectively dispersed in alcohol, facilitating the encapsulation of these drugs within ethosome. However, excessive alcohol phase content can compromise the integrity of the ethosome structure and diminish the drug encapsulation rate. Thus, selecting the appropriate alcohol phase content is important for preparing ethosome with optimal particle size and encapsulation efficiency.

**Figure 3:**
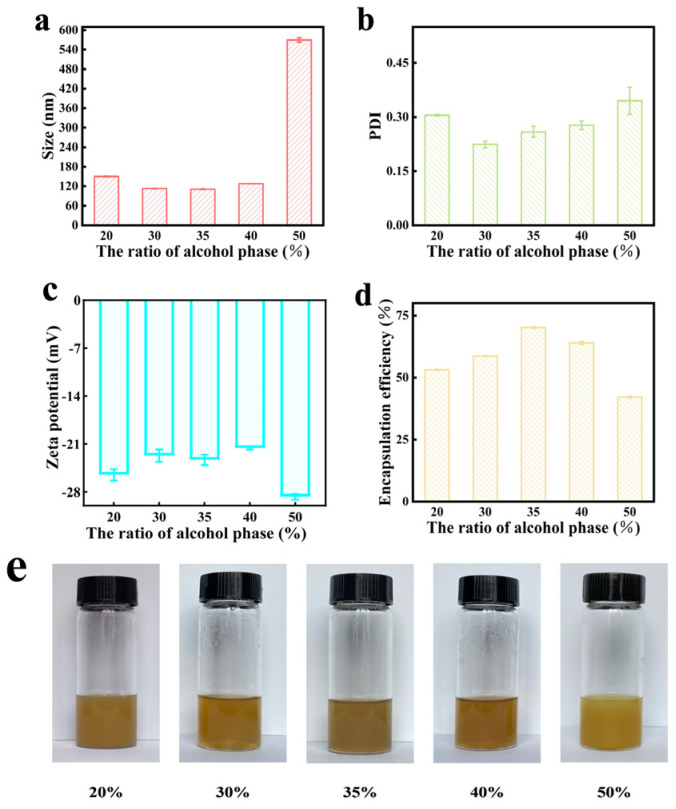
Effect of alcohol ratio on the total flavonoid ethosome of *Vernonia anthelmintica*. (**a**) Effect of alcohol ratio on size of total flavonoid ethosome. (**b**) Effect of alcohol ratio on PDI of total flavonoid ethosome. (**c**) Effect of alcohol ratio on zeta potential of total flavonoid ethosome. (**d**) Effect of alcohol ratio on encapsulation efficiency of total flavonoid ethosome. (**e**) Appearance of total flavonoid ethosome with different alcohol ratio.

Figure 3 shows that the encapsulation efficiency of alcohol-containing ethosome initially increased and subsequently decreased as the proportion of the alcohol phase rose. At a 35% alcohol phase ratio, the resulting ethosome exhibited smaller particle sizes and higher encapsulation efficiencies. In contrast, the formulation with a 50% alcohol phase proportion demonstrated a greater negative charge; however, its particle size did not meet the formulation requirements, and its encapsulation efficiency was low. This discrepancy may be attributed to the high alcohol phase proportion, which can lead to incomplete emulsification during formulation preparation, resulting in suboptimal vesicle formation and reduced drug encapsulation. As illustrated in Figure 3, all alcohol-containing ethosome, except those with a 50% alcohol phase proportion that appeared milky-white, exhibited a uniform and clear appearance. Therefore, after considering all factors, a 35% alcohol phase proportion was selected for subsequent studies.

#### 2.3.3. Effect of the Ethanol/1,2-Propanediol Ratio on Total Flavonoid Ethosome

Ethanol, 1,2-propanediol, and other low molecular weight alcohols are commonly used to form alcohol-containing ethosome structures [27]. While the exclusive use of ethanol for preparing these ethosome can enhance drug permeation, its significant volatility may lead to reduced drug retention on the skin. In contrast, propylene glycol is a colorless, viscous liquid with hygroscopic and lubricating properties, which are advantageous for prolonging the duration of phospholipid bilayer vesicles retention on the skin and increasing drug accumulation in its deeper layers. Furthermore, the combination of ethanol and 1,2-propanediol not only minimizes the quantity of ethanol required but also improves the stability of the formulation, enhances drug deposition on the skin, and maintains drug permeation. This study evaluated various dosages of ethanol and 1,2-propanediol, establishing a foundation to prepare drug-loaded alcohol-containing ethosome.

**Figure 4:**
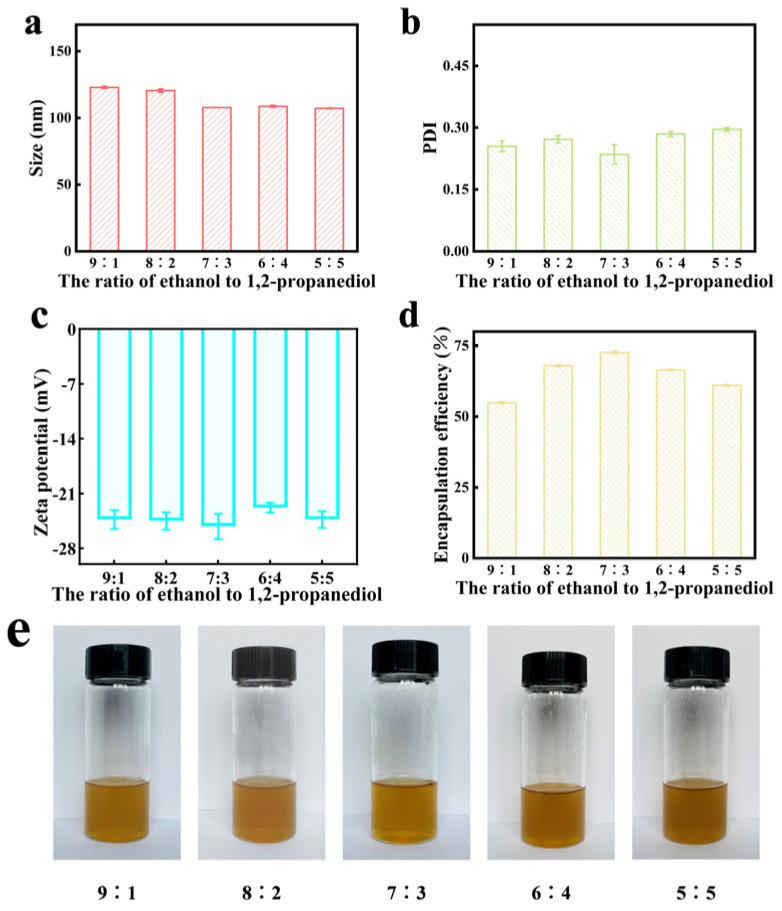
Effect of ethanol/1,2-propanediol ratio on the total flavonoid ethosome. (**a**) Effect of ethanol/1,2-propanediol ratio on size of total flavonoid ethosome. (**b**) Effect of ethanol/1,2-propanediol ratio on PDI of total flavonoid ethosome. (**c**) Effect of ethanol/1,2-propanediol ratio on zeta potential of total flavonoid ethosome. (**d**) Effect of ethanol/1,2-propanediol ratio on encapsulation efficiency of total flavonoid ethosome. (**e**) Appearance of total flavonoid ethosome with different ethanol/1,2-propanediol ratios.

Figure 4 shows that the optimal preparation conditions are achieved when the ratio of ethanol/1,2-propanediol is 7:3. Under these conditions, the drug-loaded ethosome exhibit a smaller particle size, a more uniform particle distribution, and a higher encapsulation efficiency. The absolute value of the zeta potential of the alcohol ethosome is greater, indicating enhanced stability. The appearance of the ethosome after drug loading is uniform and clear, with no observable signs of precipitation or aggregation.

#### 2.3.4. Effect of Soy Lecithin Concentration on Total Flavonoid Ethosome

Phospholipids are critical as drug excipients, functioning as nano-drug carriers to form cysts and enhance membrane fusion. These phospholipids primarily consist of natural lecithin and synthetic phospholipids. Due to their high cost and limited use in industrial production, synthetic phospholipids are rarely employed; hence, natural lecithin is typically utilized as the raw material for preparing alcohol-containing ethosome [28]. The commonly used phospholipids for formulating alcohol-containing ethosome is egg yolk lecithin and soy lecithin. Alcohol-containing ethosome formulated with egg yolk lecithin exhibit good robustness and impermeability. In comparison, soy lecithin, refined from soybean phospholipids, contains lower lipid impurities and generally yields alcohol-containing ethosome with enhanced stability. Therefore, this study selected soy lecithin as the raw material for preparing drug-loaded alcohol-containing ethosome.

Typically, the encapsulation efficiency and particle size of drug-loaded ethosome increase with higher concentrations of soy lecithin. However, excessively high concentrations can lead to increased lipid oxidation and degradation, which adversely affects the system’s stability and may promote the rapid formation of multi-chambered vesicles, ultimately resulting in decreased encapsulation efficiency. Consequently, to optimize both the skin penetration ability and encapsulation efficiency of alcohol-containing ethosome, minimizing the particle size of the prepared ethosome is crucial, making the concentration of soy lecithin in the formulation design an important consideration.

**Figure 5:**
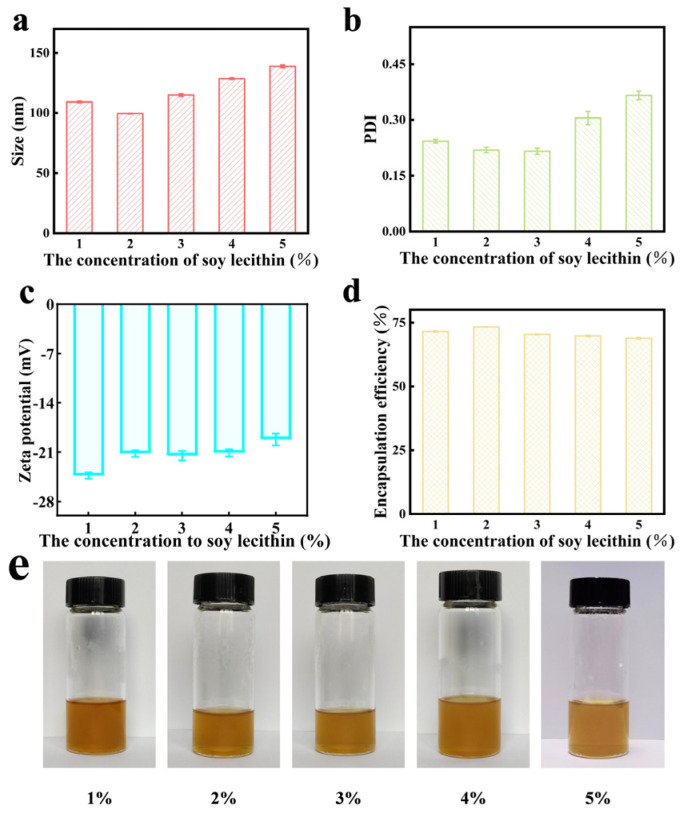
Effect of soy lecithin concentration on the total flavonoid ethosome of *Vernonia anthelmintica*. (**a**) Effect of soy lecithin concentration on size of total flavonoid ethosome. (**b**) Effect of soy lecithin concentration on PDI of total flavonoid ethosome. (**c**) Effect of soy lecithin concentration on zeta potential of total flavonoid ethosome. (**d**) Effect of soy lecithin concentration on encapsulation efficiency of total flavonoid ethosome. (**e**) Appearance of total flavonoid ethosome with different soy lecithin concentrations.

Figure 5 shows the influence of different concentrations of soy lecithin on the characterization of quercetin-loaded ethosome within the system. At a soy lecithin concentration of 2%, the drug-loaded ethosome exhibited a reduced particle size and enhanced encapsulation efficiency, with particles evenly distributed throughout the system. This indicates improved skin penetration and superior drug encapsulation by the ethosome. Conversely, when the soy lecithin concentration exceeded 2%, the particle size and PDI increased with rising lecithin concentration. Concurrently, the negative potential and encapsulation efficiency displayed a declining trend. Figure 5 shows that the ethosome loaded with total flavonoids appeared as a yellowish-clear solution with uniform consistency and no precipitation or aggregation was observed.

### 2.4. Preparation and Characterization of Total Flavonoid Ethosome from Vernonia anthelmintica (L.) Willd.

**Figure 6:**
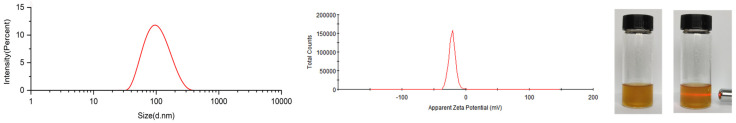
Appearance and particle size potential distribution of total flavonoid ethosome of *Vernonia anthelmintica*.

**Figure 7:**
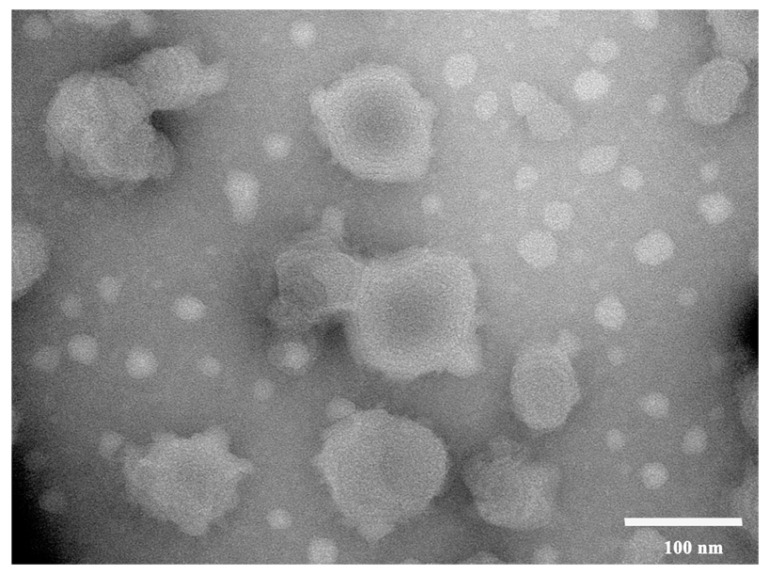
Transmission electron microscope (TEM) image of total flavonoid ethosome from *Vernonia anthelmintica* (×100 k).

Figure 6 shows the appearance, particle size, and potential distribution of drug-loaded ethosome prepared under optimized conditions. The figure illustrates that the total flavonoid ethosome of *Vernonia anthelmintica* exhibit a uniform appearance and a clear yellow solution, characterized by the Tyndall effect. The prepared drug-loaded ethosome have a particle size of 98.84 ± 0.3 nm, a PDI of 0.239 ± 0.014, and a zeta potential of −22.3 ± 0.4 mV. The encapsulation efficiency was determined to be 72.85 ± 0.42%, suggesting that a relatively stable formulation system with small particle sizes and high encapsulation efficiency was achieved, which is favorable for skin penetration. Furthermore, the results from transmission electron microscopy, as shown in Figure 7, reveal that the total flavonoid ethosome exhibit a spherical vesicle structure, with lamellar patterns discernible on the surface and a particle size of approximately 100 nm, indicating a more uniform distribution.

**Figure 8:**
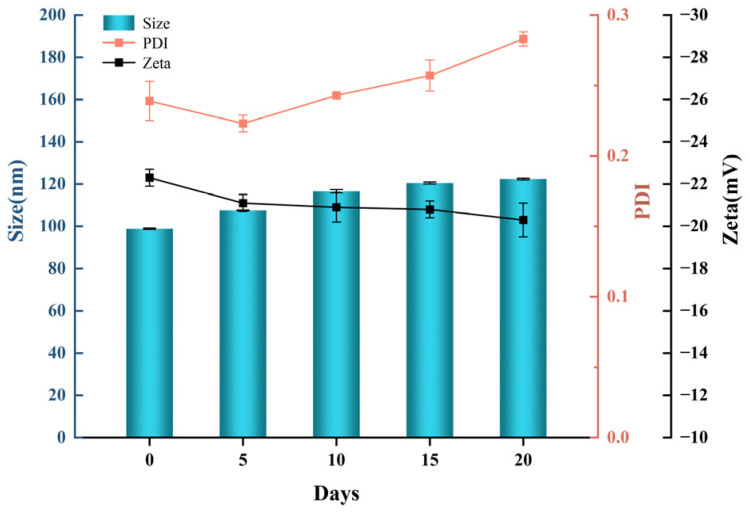
Stability study of the total flavonoid ethosome.

In order to study the stability of drug-laden ethosome, we observed the particle size, dispersion uniformity, and potentiometric values of ethosome vesicles at specific time intervals. As shown in Figure 8, the particle size of drug-laden ethosome increased with time, by about 20 nm, compared to freshly prepared formulations. This increase may be due to the adhesion of some ethosome vesicles. From day 5, the PDI gradually increased but remained below 0.3, and the negative zeta potential value of drug-laden ethosome decreased slightly, indicating a uniform distribution of ethosome vesicles and a stable structure of ethosome.

### 2.5. Preparation of Ethosome Gel with Total Flavonoids from Vernonia anthelmintica (L.) Willd.

#### 2.5.1. Screening of Gel Matrix Swelling Solution

The results of the solvent screening for the Carbomer 934 formulation are presented in Table 2. Carbomer 934 dispersed uniformly, forming a blank gel with a moisturizing sensation when swelled with water, a 40% ethanol solution, or a glycerin–propylene glycol–water solution (comprising 10% glycerin and 5% 1,2-propanediol, Gly–PG–H_2_O). The gel prepared with the 40% ethanol solution exhibited slightly lower density and a cooling sensation upon application due to the presence of ethanol, which enhanced the volatility of the formulation, reduced the viscosity of the Carbomer gel system, and introduced some potential skin irritancy. The Carbomer gel swelled with water displayed a slightly turbid transparency. Upon the addition of an appropriate amount of glycerin and 1,2-propanediol solution, a colorless and transparent gel was achieved, which enhanced the formulation’s moisturizing effect and permeation-enhancing capability. Consequently, Gly–PG–H_2_O had the highest comprehensive score. This study selected Gly–PG–H_2_O as the optimal swelling solution for the gel matrix.

#### 2.5.2. Screening of Gel pH

The Carbomer solution is inherently acidic, necessitating the addition of an alkalizing agent, such as triethanolamine, to achieve the appropriate pH for optimal gel formation and viscosity. Both excessively high and low pH values can adversely affect gel formation, thereby influencing the gel’s physical properties and the drug’s release. Consequently, pH emerges as a critical factor in the performance of Carbomer gel. The study investigated the impact of varying pH levels on the properties of the gel matrix. As indicated in Table 3, the viscosity of the Carbomer 934 dispersion initially increased with rising pH, followed by a decrease. At a pH of 10.0, the viscosity of the Carbomer 934 gel was markedly low, leading to inadequate skin retention. In contrast, the Carbomer 934 gel at pH 7.0 exhibited favorable formability, optimal viscosity, and a moisturized, delicate texture that closely resembles the pH of skin tissue. Therefore, considering the comprehensive evaluation, the pH of the gel matrix was adjusted to 7.0.

#### 2.5.3. Screening of Gel Matrix Concentration

The concentration of Carbomer is critical to the gel’s formability and viscosity, and the effect of varying Carbomer 934 concentrations on the properties of the gel matrix was investigated. Table 4 demonstrates that a Carbomer concentration of 0.1% does not allow for gel formation. As the Carbomer content increases, the viscosity of the Carbomer dispersion gradually rises. When the Carbomer concentration exceeds 0.9%, the resulting high-viscosity gel may feel heavy on the skin and can also influence the rheological behavior of the entire gel system. In contrast, a Carbomer concentration of 0.7% produces a uniformly distributed gel with moderate viscosity, which is easy to spread and offers a moisturized, delicate texture.

#### 2.5.4. Preparation, Content Determination, and Characterization of Alcoholic Plasmatic Gel

**Figure 9:**
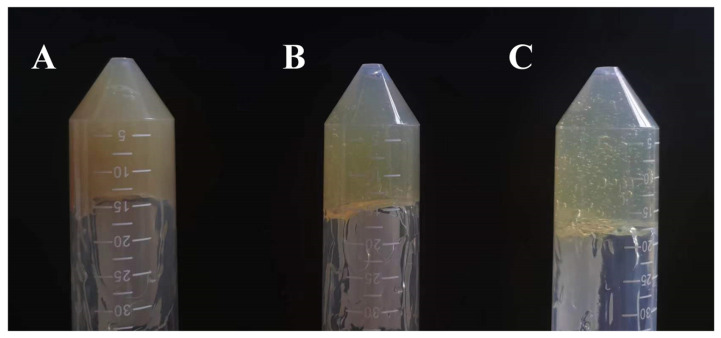
Appearance of ethosome gel with different drug/matrix ratios: (**A**) 1:1; (**B**) 1:3; (**C**) 1:6.

**Figure 10:**
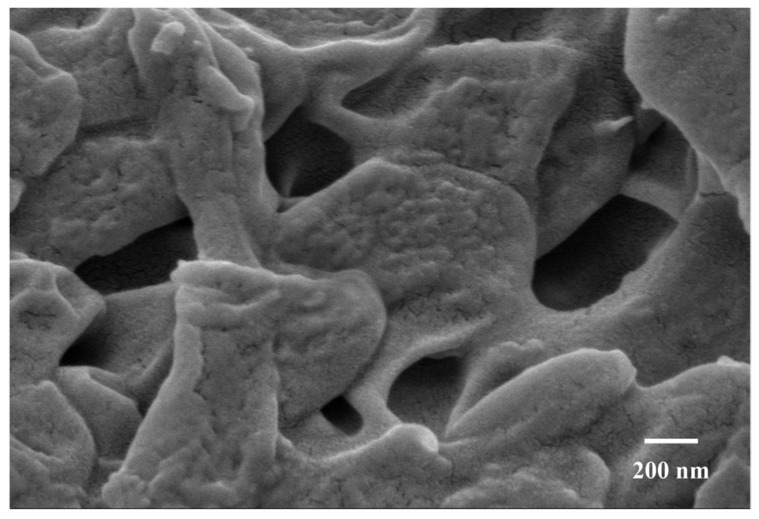
SEM characterization of ethosome gel (×100 k).

According to Table 5, the drug/matrix ratio primarily influences the color appearance of the ethosome gel, with minimal impact on its formability, viscosity, uniformity, spreadability, and skin feel. Figure 9 illustrates that ethosome gel with three distinct drug/matrix ratios exhibit a consistent texture, moderate viscosity, and lack of layering. As the blank matrix ratio increases, the color of the ethosome gel transitions gradually from yellow to light yellow. The drug/matrix ratio of 1:1 had the best composite score for quality. Regarding the dosage design, the 1:1 drug/matrix ratio was chosen to prepare the ethosome gel. Heat resistance, cold resistance, and centrifugation tests demonstrated no significant changes in the appearance of the ethosome gel, nor any layering, indicating its good stability. The drug content determination results for the ethosome gel indicated that the drug content for the three batches of samples was 0.301 mg/g, 0.303 mg/g, and 0.301 mg/g, respectively, yielding an average content of 0.302 mg/g and a relative standard deviation (RSD) of 0.484%. As illustrated in Figure 10, scanning electron microscopy (SEM) observed that ethosome gel had a distinct pore structure, which enhanced its water absorption capacity.

### 2.6. Pharmacological Investigation of Total Flavonoid Ethosome Gel

#### 2.6.1. Visualization of the Results of Animal Experiments

**Figure 11:**
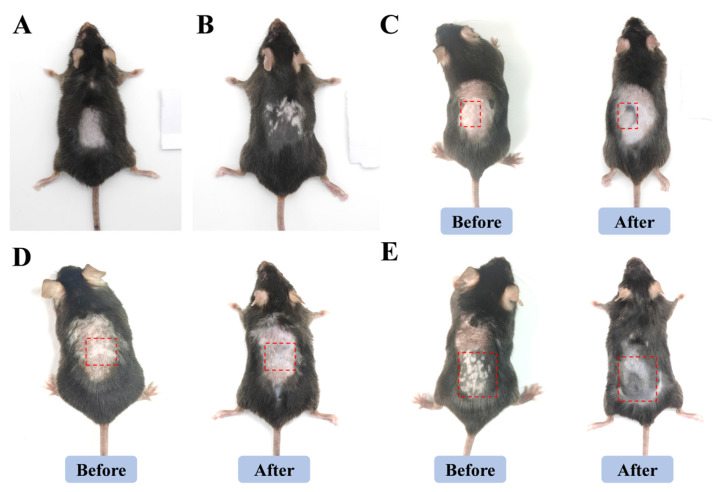
Visualization of mouse skin tissue. (**A**) Control group. (**B**) Model group. (**C**) Ethosome gel group. (**D**) Solution group. (**E**) KL Group.

There were no white patches on the skin of the blank mice (Figure 11A). Compared with the blank group, the mice in the model group had obvious white patches on the skin and white fur, indicating that the vitiligo model was successfully constructed (Figure 11B). The ethosome gel group, solution group, and kallikrein tincture group (KL) mice had reduced white patches and re-pigmented skin on their backs, with more significant treatment effects in the ethosome gel group and KL group. (Figure 11C–E). This shows that the ethosome gel formulation enhances the efficacy of the drug.

**Figure 12:**
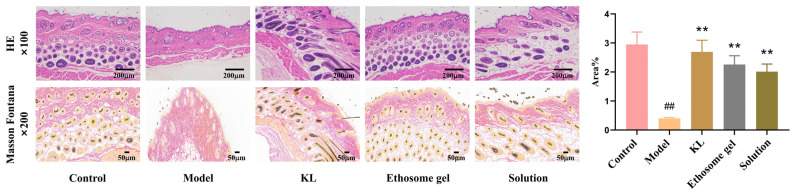
Staining results of mouse skin tissue sections and quantification of melanin. Data were evaluated using one-way ANOVA (mean ± SD, *n* = 6). ^##^ *p* < 0.01 vs. control; ** *p* < 0.01 vs. model.

Through H&E staining and Masson Fontana staining of skin tissue sections (Figure 12), it is evident that the model group exhibited the lowest melanin levels in the skin, the KL group had the highest content, and the solution and ethosome gel groups had visible therapeutic effects. The increase in melanin content in the ethosome gel group compared to the solution group proved that the ethosome gel group could improve the therapeutic effect of the drug on vitiligo.

#### 2.6.2. Animal Treatment Effects and Related Indicator Detection

**Figure 13:**
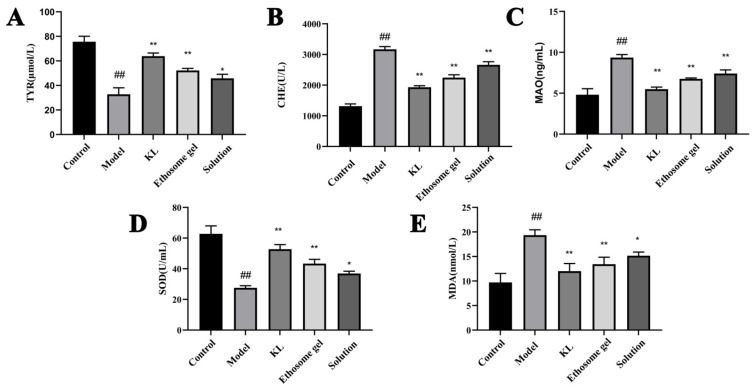
Effects of ethosome gel on serum index in model mice. (**A**) Effects of ethosome gel on TYR. (**B**) Effects of ethosome gel on CHE. (**C**) Effects of ethosome gel on MAO. (**D**) Effects of ethosome gel on SOD. (**E**) Effects of ethosome gel on MDA. Data were evaluated using one-way ANOVA (mean ± SD, *n* = 6). ^##^ *p* < 0.01 vs. control. * *p* < 0.05, ** *p* < 0.01 vs. model.

As illustrated in Figure 13, the serum index test results for mice indicate that, compared to the model group, the levels of TYR and SOD in the ethosome gel treatment group were significantly increased (*p* < 0.01). Conversely, the levels of CHE, MAO, and MDA were significantly decreased (*p* < 0.01). These findings suggest that the total flavonoids in the ethosome gel can activate tyrosinase. Additionally, the existing literature demonstrates that the antioxidant enzyme thioredoxin reductase-1 (TRXR1) controls the stability and function of MITF, the master regulator of melanocytes and melanoma. TRXR1 also plays a crucial role in regulating the body’s oxidative–antioxidative balance and mitigating the oxidative stress associated with vitiligo [29]. Compared to the reference solution, the ethosome gel group exhibited superior therapeutic effects, indicating that this transdermal formulation can enhance the stability and permeability of liquid formulations, prolong the duration of drug action, and function as an effective transdermal delivery system. The ethosome gel group also demonstrated improved melanin deposition, as evidenced by changes in serum indicators and quantitative analysis of melanin staining results, thereby underscoring the advantages of the ethosome gel formulation over traditional dosage forms in transdermal drug delivery.

## 3. Conclusions

In summary, this study developed an ethosome gel loaded with total flavonoids derived from *Vernonia anthelmintica* (L.) Willd. The formulation consisted of phospholipid double-layered spherical vesicles characterized by small particle size, uniform dispersion, and good stability. During the preparation process, a mixture of ethanol and propylene glycol was utilized as the alcohol phase, enhancing the formulation’s permeability and increasing its stability. By integrating ethosome with porous gel, the utilization of the drug was further improved. Results from animal experiments demonstrated that the formulation exhibited excellent anti-vitiligo effects by increasing tyrosinase activity and regulating the body’s antioxidant mechanisms. Intragroup comparisons with the reference stock solution underscored the advantages of transdermal drug penetration offered by ethosome gel nanoformulations. Furthermore, ethosome gel enhanced patient compliance.

## 4. Materials and Methods

### 4.1. Reagents and Equipment

*Vernonia anthelmintica* (L.) Willd., a plant of the Asteraceae family, was collected from Moyu County, Hotan Prefecture, Xinjiang Uyghur Autonomous Region. The plant material was identified by Professor Yun Zhu from the School of Pharmacy, Shihezi University, Xinjiang, China. Lutin (Solarbio, Beijing, China, purity ≥ 98%); NaOH (Yongsheng Fine Chemical Co., Ltd., Tianjin, China, analytical grade); NaNO_2_ (Yongsheng Fine Chemical Co., Ltd., Tianjin, China, analytical grade); Al(NO_3_)_3_ (Macklin Biochemical Technology Co., Ltd., Shanghai, China, analytical grade); soybean lecithin (Solarbio, Beijing, China, L8050); cholesterol (Solarbio, Beijing, China, C8280); anhydrous ethanol (HuanYu Fine Chemical Co., Ltd., Tianjin, China); 1,2-propanediol (Beilian Fine Chemical Development Co., Ltd., Tianjin, China); cellulose acetate membrane (Sartorius, Beijing, China); hydroquinone (Shuisheng Fine Chemical Co., Ltd., Tianjin, China, analytical grade); CMC-Na (Yuan Ye Biotechnology Co., Ltd., Shanghai, China, S14016); methanol (Beilian Fine Chemical Development Co., Ltd., Tianjin, China, analytical grade); and Carbomer 934 (Yuan Ye Biotechnology Co., Ltd., Shanghai, China) were purchased as described. The CHE kit, SOD kit, MDA kit, TyR kit, and MAO kit were all purchased from Jiancheng Bioengineering Institute, Nanjing, China. Glycerol was purchased from BioFroxx, Einhausen, Germany.

The following equipment was utilized: VCX 130 Ultrasonic Cell Disruptor (SONICS, Newtown, CT, USA); FE20 Plus pH Meter (Mettler-Toledo Instrument Co., Ltd., Shanghai, China); UV-2600 UV–Vis Spectrophotometer (Shimadzu, Kyoto, Japan); AREX Digital Pro Magnetic Stirrer with Heating (VELP, Usmate Velate, Italy); HH-4 Digital Thermostatic Water Bath (LCB Instrument Technology Co., Ltd., Shanghai, China); H3-18KR Tabletop High-speed Refrigerated Centrifuge (Zhaodi Biotechnology Co., Ltd., Shanghai, China); FD-2A Freeze Dryer (Bilang Instrument Manufacturing Co., Ltd., Shanghai, China); UPWS-I-10T Ultra-pure Water Purifier (Yongjieda Purification Technology Co., Ltd., Hangzhou, China); Malvern nano-particle size analyzer (Zetasizer Nano-ZS3690, Malvern Instruments LTD, Malvern, UK).

### 4.2. Animals

Thirty SPF-grade C57BL/6 male mice (20 ± 5 g) were purchased from Henan Sikebeis Biotechnology Co., Ltd. (Anyang, China). The animal production license number was SCXK (Yu) 2020-0005. The mice were kept in an environment with a temperature of 24 ± 3 °C and humidity of 55 ± 10%, with 12 h of light and free access to food and water. The animal experiment was approved by the Ethics Committee on Animal Experimentation of the First Affiliated Hospital of Shihezi University (Approval No. A2022-085-01).

### 4.3. Preparation Method of Ethosome

#### 4.3.1. Injection-Ultrasound Method

A total of 0.20 g of lecithin derived from soybeans and 0.04 g of cholesterol were thoroughly dissolved in 3.5 mL of a solvent mixture consisting of ethanol and 1,2-propanediol in a 7:3 ratio. This resulting solution was subsequently loaded into a 5 mL syringe. While maintaining magnetic stirring at a temperature of 35 °C and a rotation speed of 600 rpm, 6.5 mL of distilled water was added gradually over 1 h. The mixture was then subjected to ultrasonic disruption for 30 min in an ice bath (at 130 W, with 10 s of ultrasound followed by a 15 s pause). To obtain ethosome, the resulting suspensions were filtered through filters with pore sizes of 0.45 μm and 0.22 μm, respectively. The Malvern nanoparticle size analyzer was employed to measure the vesicle size and PDI of the produced ethosome.

#### 4.3.2. Thin Film Dispersion Technique

In a round-bottom flask, 0.20 g of soybean lecithin and 0.04 g of cholesterol were accurately measured. To dissolve the lipids, 3 mL of chloroform was introduced and mixed by shaking. The organic solvent was subsequently evaporated under reduced pressure, yielding a uniform thin film. Following this, a mixture of 3.5 mL of ethanol/1,2-propanediol (with a ethanol/1,2-propanediol ratio of 7:3) and 6.5 mL of distilled water was added to the flask. Continuous shaking ensured that the thin film completely detached, resulting in a homogenous milky-white dispersion. This mixture was then subjected to ultrasonic disruption (130 W, with 10 s of ultrasound followed by a 15 s interval) for 30 min in an ice bath. Finally, the suspensions were filtered using 0.45 μm and 0.22 μm filters to isolate the ethosome. The particle size and PDI of the obtained ethosome were assessed using a Malvern nanoparticle size analyzer.

### 4.4. Selection of Alcohol Phase

Ethanol, along with 1,2-propanediol and additional short-chain alcohols, was employed in the formulation of ethosome. Ethanol improves the flexibility of lipid membranes, which facilitates transdermal absorption, whereas 1,2-propanediol enhances the formulation’s stability and aids in drug permeation and absorption. As a result, this research produced ethosome using ethanol, 1,2-propanediol, and a combination of both ethanol and 1,2-propanediol, as detailed in Section 4.3.1. The effects of different alcohol phases on vesicle size, PDI, zeta potential, and various other attributes of the ethosome were examined.

### 4.5. Total Flavonoid Entrapment Efficiency of the Prepared Ethosome (EE%)

The encapsulation efficiency (EE%) of the prepared ethosome was assessed via a low-temperature, high-speed centrifugation technique. A volume of 1.0 mL of freshly formulated drug-loaded alcoholic vesicles was carefully placed into a centrifuge tube and spun at 14,000 r/min for 30 min at a temperature of 4 °C. The supernatant derived from blank ethosome was utilized as the reference solution, to which sequential additions of 5% NaNO_2_, 10% Al(NO3)_3_, and 4% NaOH were made for color development. After allowing the mixture to rest for several minutes, the concentration of the unencapsulated drug was quantified at 504 nm and recorded as W_1_. Following this, 1.0 mL of the drug-loaded ethosome suspension was transferred into a 10 mL volumetric flask, after which methanol was introduced, and the mixture was subjected to ultrasonic treatment for 20 min to break down the ethosome. The supernatant from the blank disrupted ethosome, treated similarly, served as the reference solution to determine the total drug amount present in the ethosome, which was documented as W_2_. The encapsulation efficiency was subsequently calculated using the following formula:

$$\mathrm{EE}(\%)=(1-\frac{W_{1}}{W_{2}})\times 100\%$$

### 4.6. Optimization of Prescription for Total Flavonoid Ethosome

#### 4.6.1. Impact of Cholesterol/Soybean Lecithin Ratio on Total Flavonoid Ethosome

The amounts of cholesterol and soybean lecithin were measured to achieve cholesterol/soy lecithin ratios of 1:2, 1:3, 1:5, 1:8, and 1:15. A total of 6 mg of purified total flavonoids was measured out and dissolved in 4 mL of a mixture of ethanol and 1,2-propanediol (ethanol/1,2-propanediol ratio of 4:1). This mixture was then transferred into a 5 mL syringe and added gradually into 6 mL of distilled water while being magnetically stirred at a temperature of 35 °C and a speed of 600 rpm. After one hour, the resulting suspensions underwent ultrasonic treatment in an ice bath using a cell disruptor set to 130 W, with ultrasonic application for 10 s followed by a 15 s rest period, for 30 min. The resulting solutions were subsequently filtered using 0.45 μm and 0.22 μm membranes. The encapsulation efficiency, particle size, and PDI were then assessed. The zeta potential was evaluated after diluting the samples with distilled water at 1:20.

#### 4.6.2. Influence of Alcohol Phase Ratio on Total Flavonoid Ethosome

In a beaker, 6 mg of purified total flavonoids was combined with 0.20 g of soybean phospholipids and 0.04 g of cholesterol. Sequentially, ethanol/1,2-propanediol solutions (ethanol/1,2-propanediol ratio of 4:1) with ratios of 20%, 30%, 35%, 40%, and 50% were introduced into the mixture. Once dissolved, the aqueous phase was gradually added while stirring magnetically at 35 °C and 600 rpm. After an hour, the resulting suspensions were subjected to ultrasonic treatment in an ice bath with a cell disruptor (130 W, 10 s of ultrasonic treatment followed by 15 s of rest) for 30 min. The mixtures were then filtered using 0.45 μm and 0.22 μm membranes. Post-treatment encapsulation efficiency was measured, along with the particle size, PDI, and zeta potential, calculated after diluting with distilled water at a factor of 20.

#### 4.6.3. Effect of Ethanol/1,2-Propanediol Ratio on Total Flavonoid Ethosome

A total of 0.20 g of soybean phospholipids, 0.04 g of cholesterol, and 6 mg of purified total flavonoids were incorporated into a solution comprising 3.5 mL of ethanol/1,2-propanediol, with varying ethanol/1,2-propanediol ratios of 9:1, 8:2, 7:3, 6:4, and 5:5. Following this, 6.5 mL of distilled water was added slowly while maintaining magnetic stirring at a temperature of 35 °C and a speed of 600 rpm. After stirring for one hour, the resulting suspensions were subjected to ultrasonic treatment in an ice bath using a cell disruptor (130 W, ultrasonic for 10 s with a 15 s pause) for 30 min. The mixtures obtained were then filtered through membranes with pore sizes of 0.45 μm and 0.22 μm. The encapsulation efficiency was evaluated, and the sample’s particle size, PDI, and zeta potential were assessed after being diluted 20-fold with distilled water. In a subsequent experiment, 6 mg of purified total flavonoids was combined with a suitable amount of soy lecithin and cholesterol in a ratio of 5:1. The soy lecithin concentration was varied at 1%, 2%, 3%, 4%, and 5%. These components were dissolved in a 3.5 mL ethanol/1,2-propanediol mixture (7:3 ethanol/1,2-propanediol ratio) while stirring magnetically at 35 °C at a rate of 600 rpm. After gradually adding 6.5 mL of distilled water, the mixture was hydrated for one hour. Ultrasonic treatment was again applied to the suspensions using a cell disruptor (130 W, ultrasonic for 10 s with a 15 s interval) for 30 min in an ice bath. The final mixtures were filtered through 0.45 μm and 0.22 μm membranes. The encapsulation efficiency was calculated, and measurements of the particle size, PDI, and zeta potential were made after diluting the samples 20 times with distilled water.

### 4.7. Preparation and Characterization of Total Flavonoid Ethosome from Vernonia anthelmintica (L.) Willd.

After thoroughly evaluating prescription factors, the injection-ultrasound technique was utilized to fabricate drug-loaded alcohol chitosan nanoparticles. The process of preparation began with the weighing and complete dissolution of 0.20 g of soy lecithin, 0.04 g of cholesterol, and 6 mg of purified total flavonoids in 3.5 mL of an ethanol/1,2-propanediol mix (with a ratio of 7:3) to create the alcohol phase. Subsequently, 6.5 mL of distilled water served as the aqueous phase, into which the alcohol phase was gradually introduced at 35 °C while stirring at 600 rpm. Following one hour of hydration, the mixture experienced cell disruption and ultrasonication in an ice bath (130 W, 10 s of ultrasound followed by a 15 s pause) for 30 min. The resultant suspension was filtered using 0.45 μm and 0.22 μm filters to yield the final product.

The optimized formulation of flavonoid-loaded alcohol chitosan nanoparticles was characterized through various assessments: 1. Visual assessment: documenting the appearance by capturing images. 2. Determining encapsulation efficiency: performed per the method described in Section 4.5. 3. Measurement of particle size, PDI, and zeta potential: after diluting with pure water by a factor of 20, readings were obtained using a Malvern Nano Particle Size Analyzer. 4. Morphological analysis: 20 μL of drug-loaded alcohol chitosan nanoparticles were applied to a copper grid, stained negatively with 2% phosphotungstic acid, dried, and examined under a transmission electron microscope (TEM) for morphology evaluation and imaging. 5. Stability assessment: the formulated drug-loaded alcohol chitosan nanoparticles were stored in a centrifuge tube, sealed with film to inhibit volatilization, and maintained at 4 °C. Samples were collected on days 0, 5, 10, 15, and 20 to measure particle size, PDI, and zeta potential, thereby investigating their stability during storage.

### 4.8. Preparation of Ethosome Gel Containing Flavonoids of Vernonia anthelmintica (L.) Willd.

#### 4.8.1. Selection of Gel Matrix

Carbomer and various temperature-sensitive matrices, cellulose derivatives, chitosan, and other pH-sensitive formulations are frequently employed in modern gel preparation efforts. Gel that respond to temperature changes show significant sensitivity to variations in temperature, and their transition temperature can be readily managed, making them suitable for applications in managing pathological fever and induced hyperthermia. In contrast, pH-sensitive gel reach their peak viscosity and texture within a pH range of 6 to 11, facilitating drug retention on the skin. Notably, Carbomer 934 serves as a water-soluble matrix distinguished by its lack of foreign body sensation, non-greasy texture, and remarkable stability; hence, this research opted for Carbomer 934 as the preferred gel matrix.

#### 4.8.2. Gel Matrix Swelling Solution Screening

Carbomer solutions were formulated using typical solvents such as water and ethanol, along with humectants like glycerol and 1,2-propanediol, to reduce irritation and enhance transdermal absorption. Evaluations were conducted on gel’s appearance, viscosity, consistency, and skin feel to determine appropriate swelling solutions for Carbomer 934. For this, three samples of Carbomer 934, each with a mass of 0.1 g, were combined with 20 mL of water, 20 mL of a 40% ethanol solution, and 20 mL of a glycerol–1,2-propanediol–water blend (containing 10% glycerol and 5% 1,2-propanediol), respectively. The mixtures were permitted to rest at room temperature for 24 h to ensure complete swelling of the Carbomer 934. Following this, a dropwise addition of 20% triethanolamine was performed while stirring until the pH reached 6.7, resulting in a blank gel matrix.

#### 4.8.3. Screening of Gel

An amount of 0.1 g of Carbomer 934 was uniformly distributed into a mixture of glycerol–1,2-propanediol–water (comprising 10% glycerol and 5% 1,2-propanediol) inside a 20 mL container. This combination was allowed to sit at ambient temperature for 24 h to ensure full swelling. Following this, a 20%triethanolamine solution was added gradually while stirring, aiming to adjust the pH to 6.0, 7.0, 8.0, 9.0, and 10.0, which produced blank gel with differing pH levels. The gel were subsequently assessed for formability, viscosity, uniformity, spreadability, skin feel, and visual appearance.

#### 4.8.4. Selection of Gel Matrix Concentration

A precise quantity of Carbomer 934 was evenly integrated into a 20 mL formulation of glycerol–1,2-propanediol–water (maintaining 10% glycerol and 5% 1,2-propanediol) to achieve concentrations of 0.1%, 0.3%, 0.5%, 0.7%, 0.9%, 1.1%, and 1.3%. After being left at room temperature for 24 h, triethanolamine at a concentration of 20% was gradually introduced while stirring, allowing for pH adjustment to 7.0, resulting in the formation of blank gel matrices with various concentrations. The resulting gel underwent evaluation for properties such as molding capabilities, viscosity, uniformity, spreadability, skin feel, and appearance.

#### 4.8.5. Preparation, Content Determination, and Characterization of Ethosome Gel

To create drug/matrix ratios of 1:1, 1:3, and 1:6, different volumes of ethosome suspensions were combined with the matrix. The pH was adjusted to 7.0 by adding a few drops of 20% triethanolamine, facilitating the evaluation of properties such as molding characteristics, viscosity, uniformity, spreadability, skin feel, and gel appearance. Three sample batches were prepared, with each sample weighing precisely 3.0 g. These samples were dissolved in methanol, thoroughly shaken, and subjected to sonication for 30 min to ensure complete mixing. The mixtures were then centrifuged at 8000 rpm for 10 min at a temperature of 4 °C. Following this, 2 mL of the supernatant was carefully transferred to a 10 mL volumetric flask. A control blank gel matrix, which excluded the ethosome gel, was used to assess the drug content within the ethosome gel matrix. The characterization of the morphology of these gel matrices occurred post-freeze-drying. The ethosome gel matrices were kept at temperatures of 60 °C and −20 °C for 24 h, after which they were allowed to stabilize at room temperature. After centrifugation at 3000 rpm for 30 min, stratification was noted.

#### 4.8.6. Gel Quality Evaluation Criteria

According to the 2020 edition of the Pharmacopoeia of the People’s Republic of China, a gel agent should be homogeneous and delicate, stay gelatinous at room temperature, and not become dry or liquefied [30]. In this study, the quality of the gel was evaluated based on formability, viscosity, homogeneity, spreadability, skin feel, and appearance, and was scored according to Table 6. The higher the score, the better the quality of the gel.

### 4.9. Pharmacological Investigation of Total Flavonoid Ethosome Gel

#### 4.9.1. Animal Experiment Grouping and Modeling

Thirty male C57BL/6 mice were randomly divided into five groups: the control group, model group, KL group, ethosome gel group, and solution group, with six in each group. The back hair of all mice was shaved using an electric razor. Twenty-four hours after depilation, 2.5% hydroquinone was applied to the depilated location on the back of the mice to establish a vitiligo model, except for the control group. For the control group, the ethosome gel without drug was given. The model group modeled only without the drug. For the KL group, each mouse received 12.8 mL/kg/day of tincture of complex kinin. For the ethosome gel group, mice were given a dose of 0.5 g/dose twice a day. For the solution group, mice were given solution 1 mL/day at a concentration of 0.15 mg/mL twice daily. The skin was observed every three days for a total of 60 days.

#### 4.9.2. Animal Treatment and Related Indicator Detection

After the final treatment, the mice were subjected to fasting but had unrestricted access to water. Blood samples were drawn from the eye 24 h post-treatment and kept on ice for 30 min. Following centrifugation at 4 °C for 10 min at 3000 rpm, the serum was isolated and kept in a refrigerator at 4 °C for future analyses. Upon euthanasia, a 1 cm × 1 cm segment of skin from the center of the treated area was preserved in a 4% paraformaldehyde solution, embedded in paraffin, and subsequently stained with H&E and Masson Fontana.

The serum samples were analyzed using assay kits for TYR, CHE, MAO, SOD, and MDA, adhering to the protocols provided by the manufacturer. Each experiment was repeated in triplicate for every group. Statistical evaluations between groups were performed utilizing *t*-tests and one-way ANOVA, with the Tukey test applied to examine homogeneity of variance. Data visualization and analysis were carried out using GraphPad Prism 6.0 (San Diego, CA, USA), denoting statistical significance with * and ^#^ (*p* < 0.05).

## Figures and Tables

**Table 1:** Results of size, PDI, and zeta potential of three ethosomes (*n* = 3).

Alcohols	Size (nm)	PDI	Zeta Potential (mV)
Ethanol	106.60 ± 0.76	0.208 ± 0.006	−16.3 ± 0.5
1,2-Propanediol	87.86 ± 0.18	0.249 ± 0.008	−14.8 ± 0.7
Mixture	73.16 ± 0.22	0.190 ± 0.012	−14.7 ± 0.4

**Table 2:** Screening results for gel matrix swellings.

Solvent	Appearance	Viscosity	Homogeneity	Skin Feel	Comprehensive Score
Water	Slightly turbid	Suitable	Uniform	Hydrated	3.5
40% ethanol	Colorless and transparent	Slightly dilute	Uniform	Hydrated and refreshing	4
Gly–PG–H_2_O	Colorless and transparent	Suitable	Uniform	Hydrated and fine texture	5

**Table 3:** Screening results for gel pH.

pH	Formability	Viscosity	Homogeneity	Spreadability	Skin Feel	Appearance	Comprehensive Score
6.0	Forming	Slightly dilute	Uniform	Easy to spread	Hydrated	Colorless and transparent	4
7.0	Forming	Suitable	Uniform	Easy to spread	Hydrated and fine texture	Colorless and transparent	4.8
8.0	Forming	Slightly sticky	Uniform	Easy to spread	Hydrated and fine texture	Colorless and transparent	4.5
9.0	Forming	Slightly dilute	Uniform	Easy to spread	Hydrated	Colorless and transparent	3.8
10.0	Forming	Very dilute	Uniform	Easy to apply	Hydrated	Colorless and transparent	3.8

**Table 4:** Screening results for gel matrix concentration.

Carbomer 934 Content	Formability	Viscosity	Homogeneity	Spreadability	Skin Feel	Appearance	Comprehensive Score
0.1%	Unshaped	Very dilute	Uniform	Easy to apply	Hydrated	Colorless and transparent	3
0.3%	Forming	dilute	Uniform	Easy to spread	Hydrated	Colorless and transparent	4.2
0.5%	Forming	Slightly dilute	Uniform	Easy to spread	Hydrated and fine texture	Colorless and transparent	4.3
0.7%	Forming	Suitable	Uniform	Easy to spread	Hydrated and fine texture	Colorless and transparent	4.8
0.9%	Forming	Slightly sticky	Uniform	Easy to spread	Slightly heavy texture	Colorless and transparent	3.8
1.1%	Forming	Sticky	Uniform	Easy to spread	Heavy texture	Colorless and transparent	3.7
1.3%	Forming	Very sticky	Uniform	Easy to spread	Heavy texture	Colorless and transparent	3.2

**Table 5:** Effect of drug/matrix ratio on ethosome gel.

Drug/Matrix Ratio	Formability	Viscosity	Homogeneity	Spreadability	Skin Feel	Appearance	Comprehensive Score
1:1	Forming	Suitable	Uniform	Easy to spread	Hydrated	Yellow	4.3
1:3	Forming	Suitable	Uniform	Easy to spread	Hydrated	Light yellow translucent	4
1:6	Forming	Suitable	Uniform	Easy to spread	Hydrated	Pale yellow translucent	3.8

**Table 6:** Gel quality evaluation criteria table.

Score	Formability	Viscosity	Homogeneity	Spreadability	Skin Feel	Appearance
0	Unshaped	Very dilute or Very sticky	Not uniform	Hard to spread	Hydrated and heavy texture	Colorless and transparent
1–3	Part of the forming	Dilute or sticky	Partly uniform	Spread and poor delicacy	Hydrated and slightly heavy texture	Yellow–translucent
4–5	Forming	Suitable	Uniform	Easy to spread	Hydrated and fine texture	Yellow

## Data Availability

The original contributions presented in the study are included in the article, and further inquiries can be directed to the corresponding authors.
